# Interferon regulatory factor 5: a potential target for therapeutic intervention in inflammatory diseases

**DOI:** 10.3389/fimmu.2025.1535823

**Published:** 2025-03-27

**Authors:** Xinyuan Yu, Ata Ur Rehman, Lihong Dang, Xu Zhang, Jia Liu, Xiaoxing Xiong, Gang Chen, Zhihong Jian

**Affiliations:** ^1^ Department of Neurosurgery, Renmin Hospital of Wuhan University, Wuhan, Hubei, China; ^2^ Multidisciplinary Brain Protection Program (MBPP), Department of Anesthesiology, Duke University Medical Center, Durham, NC, United States; ^3^ Department of Neuroscience, Yale School of Medicine, New Haven, CT, United States

**Keywords:** IRF5, tumorigenesis, inflammatory diseases, cytokines, autoimmune diseases

## Abstract

Interferon regulatory factor 5 (IRF5) is a critical transcription factor in the IRF family, playing a pivotal role in modulating immune responses, particularly within the innate immune system. IRF5 regulates the expression of type I interferons (IFNs), proinflammatory cytokines, and other immune-related genes, essential for effective host defense against infections and immune surveillance. Its functions, however, are diverse and highly context-dependent, adapting to different immune challenges and tissue environments. Studies have demonstrated that dysregulated IRF5 activation contributes to the pathogenesis of numerous diseases, including cancer, autoimmune disorders, and chronic inflammatory conditions such as systemic lupus erythematosus (SLE) and rheumatoid arthritis (RA). This dysregulation underscores the dual role of IRF5, both in immune protection and in driving pathological inflammation. Given its significant involvement in both physiological and pathological processes, IRF5 presents a promising therapeutic target for managing diseases characterized by excessive inflammation and immune dysregulation. However, developing effective molecules to specifically modulate the IRF5 pathway remains challenging, with limited therapeutic agents available for clinical application. In this review, we examine the diverse roles of IRF5 in various disease contexts, the mechanisms by which IRF5 contributes to disease progression, and the potential therapeutic strategies targeting IRF5. Additionally, we discuss potential complications and risks associated with IRF5-targeted therapies, including the balance between dampening pathological inflammation and preserving essential immune functions. This exploration highlights both the therapeutic potential and the complexity of modulating IRF5 activity in clinical settings.

## 1 Introduction

The IRF (interferon regulatory factor) family of transcription factors is essential not only in regulating gene expression and apoptosis but also in modulating cell cycle processes and tumorigenesis (1). This family comprises nine members (IRF1–IRF9), all characterized by a conserved multi-domain structure (2). Each IRF contains an N-terminal DNA-binding domain (DBD), which recognizes specific DNA sequences in interferon-stimulated response elements (3), and a C-terminal IRF-associated domain (IAD), facilitating interactions with other IRFs and proteins to form transcriptional complexes (4).

Among these, IRF5 is pivotal in regulating immune responses, including interferon expression, cellular maturation, differentiation, apoptosis, and the production of proinflammatory cytokines (5). IRF5 is constitutively expressed across various immune cell types, including dendritic cells (DCs), macrophages, and neutrophils (6, 7). The IRF5 gene in humans consists of nine coding exons and at least four alternative non-coding exons (1A, 1B, 1C, and 1D), enabling the production of multiple functional isoforms (5, 8). These isoforms, denoted as V1–V11, are expressed in a cell type-specific manner, each with distinct localization and functions (9). For instance, IRF5 isoforms V2, V9, and V10, which include exon 1B, are linked to increased susceptibility to systemic lupus erythematosus (SLE), whereas IRF5 lacking exon 1B does not confer the same risk (10).

Typically, IRF5 remains inactive until it undergoes post-translational modifications, leading to homodimerization and nuclear translocation. In the nucleus, IRF5 interacts with the NF-κB p65 RelA subunit (11), enabling it to polarize macrophages to M1 or M2 phenotypes and drive the expression of proinflammatory cytokines, such as IL-6, IL-12, and TNF-α (6, 7, 12). This activity is crucial for an effective immune response to pathogens and injuries. However, dysregulated IRF5 activation is implicated in the pathogenesis of cancer, autoimmune systems, and inflammatory diseases. This review will summarize IRF5’s roles in these conditions and examine the therapeutic potential of IRF5-targeted interventions.

## 2 Activation and function of IRF5

Extensive research demonstrates that IRF5 participates in numerous signaling pathways via activation mechanisms like ubiquitination and phosphorylation. Multiple phosphorylation sites have been identified in serine clusters at IRF5’s C-terminus, including T10, S158, S309, S317, S451, S462, S425, S427, S430, and S436 (13–15). These phosphorylation sites serve distinct functions in IRF5 activity. Specifically, S451 and S462 are pivotal for nuclear translocation, transcriptional regulation, and apoptosis (13). Phosphorylation at S436 aids in stabilizing the activated dimer, while modifications at S425, S427, and S430 are crucial for releasing the C-terminal autoinhibitory conformation. High-throughput kinase inhibitor libraries offer a valuable approach for identifying potential candidate kinases. Several kinases have been implicated in IRF5 phosphorylation, including TBK-1, TRAF6, and RIP2, with RIP2—NOD2’s downstream kinase—serving as a potent activator. In human macrophages, NOD2-induced IRF5 phosphorylation activates Akt2, enhancing glycolysis and promoting cytokine expression and macrophage polarization (16).

Ubiquitination, mediated by the E3 ubiquitin ligase TRAF6, also promotes IRF5’s nuclear translocation and binding to IFNA4, IFNA13, and IFNB promoters, though polyubiquitination itself is not essential for IRF5’s transcriptional role. Phosphorylation and ubiquitination act independently in regulating IRF5 function (13, 17).

The TLR-MyD88 pathway is well-characterized in IRF5 activation. Ligand binding to TLRs (PAMPs) leads to TLR dimerization and MyD88 recruitment, initiating downstream interactions where IRF5 associates with TRAF6 and MyD88 to activate transcription factors like NF-κB and IRF5 itself (12, 17). Additionally, TLR7/8 stimulation in human monocytes triggers IRAK4-mediated phosphorylation of IRF5 via the IRAK4-TAK1-IKKβ signaling cascade (18). Upon activation, IRF5 forms homodimers through its C-terminal dimerization domain, translocate to the nucleus, and binds to gene promoters in coordination with coactivators. Studies also show that IRF5 modulates IFN-β production in response to C. albicans through the Dectin-1-Syk-IRF5 pathway in dendritic cells (19). Additionally, DNA-damaging agents like CPT and various viral infections, including herpesviruses, can induce IRF5 phosphorylation and transcriptional activation (14, 20–23).

Upon activation and nuclear translocation, IRF5 regulates target genes essential for immune responses by coordination with coactivators. For example, it modulates the expression of pro-inflammatory cytokines, including IL-6, TNF-α, and IL-12, by recruiting the target genes through its interaction with the NF-κB subunit RelA. This supports inflammatory responses and enhances pathogen defense (6, 7, 12). Furthermore, IRF5 modulates chemokines like CXCL10 and CCL5, crucial for immune cell recruitment to infection or inflammation sites (24), and enhances the expression of co-stimulatory molecules, such as CD40 and CD86, on antigen-presenting cells, which are essential for T-cell activation and immune regulation (25).

## 3 IRF5’s unique characteristics versus IRF family members

### 3.1 IRF5 vs. IRF3/IRF7

IRF3 and IRF7 release type I interferons in response to viral infections, while IRF5 boosts pro-inflammatory cytokines such TNF-α, IL-6, and IL-12 (12). IRF5 binds to the promoters of inflammatory genes, while IRF3 and IRF7 trigger IFN-β transcription. IRF7, which shares a close association with IRF3, is a transcription factor that requires activation. IRF7 activation requires the virus-activated domain in human IRF7A (26). IRF7 is inherently located in the cytoplasm, and it is mostly expressed in B cells, plasmacytoid dendritic cells (pDCs), and monocytes in the spleen, thymus, and peripheral blood leukocytes (27, 28). Numerous stimuli, such LPS (Lipopolysaccharide), IFN-bet, EBV-latent membrane protein 1 (EBV-LMP1), viral infections, and certain chemical agents like phorbol myristate acetate (PMA) and sodium butyrate, can significantly induce IRF7 synthesis within specific cell lines (28–30). Furthermore, DNA damage, virus infection, and EBV-LMP1 can cause IRF7 phosphorylation and nuclear translocation (26, 30, 31).

### 3.2 IRF5 vs. IRF4/IRF8

During lymphocyte development, IRF4 and IRF8 operate as transcriptional repressors or activators. IRF4 regulates T-cell activity, whereas IRF5 promotes M1 macrophage polarization and inflammatory responses (32). IRF4 is only expressed in immune system cells and responds to a variety of mitogenic stimuli, such as PMA/Ionomycin and T-cell receptor (TCR) cross-linking (33–35). IRF4’s function as a transcriptional activator or suppressor is governed by its interactions with various transcription factors or the DBD on different promoters (36, 37). The human IRF8 gene is located on chromosome 16q24.1, spanning 23 kb and including 9 exons and 8 introns. The IRF8 protein encodes 426 amino acids. IRF8 is primarily found in lymphoid and myeloid cell lines, but it can also be found in the colon, skin, lung, liver, ocular lens, cornea, and heart epithelial cells. IFN-γ stimulates its expression (38–40). IRF8 is abundantly expressed in both progenitor and mature cells within the B cell, conventional DC1 (cDC1), and pDC lineages, and it plays a critical role in their development and activity.

### 3.3 RF5 vs. IRF1/IRF2

IRF1 is a tumor suppressor that regulates apoptosis and the cell cycle, but IRF5 can suppress tumors and cause cancer based on its biological context (41). The IRF1 gene is expressed at low baseline levels in human cells but can be activated by various stimuli, including IFNs, tumor necrosis factor (TNF), and interleukin-1 (IL-1) (42). Many cell types constitutively produce IRF2, and viruses and IFN can further stimulate its expression.

Building on the structural features of IRF5, including its activation, dimerization, nuclear translocation, and interactions with co-activators, targeting specific binding sites on IRF5 presents a potential avenue for therapeutic intervention. Identifying crucial peptide sequences involved in IRF5–protein interactions is essential. The development of specific small-molecule inhibitors to disrupt these processes offers a promising strategy for precise modulation of IRF5 activity, which will be elaborated on in the following section.

## 4 IRF5 and diseases

IRF5 plays a critical role in microbial resistance, cell survival, and innate immunity (43, 44), however, dysregulation of IRF5 activation is involved in various diseases including tumors, autoimmune and inflammatory disorders.

### 4.1 IRF5 and autoimmune diseases

High IRF5 expression is closely associated with several autoimmune diseases, notably systemic lupus erythematosus (SLE), multiple sclerosis (MS), Sjögren’s syndrome, and rheumatoid arthritis (RA), with particularly strong correlations in SLE and Sjögren’s syndrome (45–49). Extensive research into IRF5’s role in SLE pathogenesis has highlighted elevated inflammatory cytokines and type I interferons (IFNs) as characteristic in SLE patients (46, 50, 51). IRF5 shows high expression in peripheral blood mononuclear cells (PBMCs) of SLE patients and is persistently activated in monocytes (52, 53). Specifically, overexpressed IRF5 isoforms (v2, v9, v10) are linked to increased SLE susceptibility (10). suggesting that IRF5 dysregulation may drive SLE pathogenesis. Recent genome-wide association studies have identified several SLE-risk single-nucleotide polymorphisms (SNPs) enriched in the IRF5 locus. Among these, rs4728142 could act as an enhancer to regulate the expression of IRF5 by affecting the binding affinity of zinc finger and BTB domain-containing protein 3 (ZBTB3), a transcription factor involved in regulation. Furthermore, in monocytes from SLE patients, CRISPR-based interference with the regulation of this enhancer attenuated the production of disease-associated cytokines (54).

Research shows that either genetic IRF5 deficiency (Irf5^–/–^) or pharmacological inhibition using N5-1, which blocks IRF5 nuclear translocation, effectively protects against SLE onset and severity in murine lupus models (46, 51, 55, 56). Additionally, IRF5 deficiency shields mice from inflammatory damage in both inflammatory and lupus arthritis models (48, 55, 57–59). In antigen-induced arthritis models, Irf5^–/–^ mice show reduced knee swelling and lower levels of proinflammatory cytokine IL-12p40 (57). Further, IRAK4 inhibition, which downregulates IRF5, alleviates joint inflammation in RA by reducing IRF5 activity in macrophages and fibroblasts (60).

### 4.2 IRF5 role in inflammatory signaling and autoimmune diseases

The IRF family has nine members (IRF1-IRF9), each with a unique role in immunological regulation, inflammation, and oncogenesis. While numerous IRFs, including IRF3, IRF4, IRF7, and IRF8, have been linked to these processes, IRF5 stands out for its distinct role in increasing the production of pro-inflammatory cytokine. IRF5 is a transcriptional regulator of inflammatory pathways activated through Toll-like receptors (TLRs), leading to the production of cytokines such as TNF-α, IL-6, and IL-12 (12), unlike IRF3 and IRF7, which primarily drive antiviral responses via type I interferon (IFN) signaling (Honda et al., 2006). This renders IRF5 especially important in the development of autoimmune illnesses such as rheumatoid arthritis and systemic lupus erythematosus (SLE) in which increased inflammatory cytokine production drives disease progression (10). Conversely, IRF4 suppresses the polarization of inflammatory macrophage, demonstrating IRF5’s specific pro-inflammatory activity (61).

IRF5 also plays a distinct and context-specific role in cancer, distinguishing it from other IRFs that act primarily as tumor suppressors or oncogenes. Such as IRF1 and IRF8 support the suppression of tumors by promoting apoptosis and improving immune control (41), but IRF5 has a coupled role based on the tumor microenvironment as well. In some malignancies, including breast cancer, IRF5 acts as a tumor suppressor by triggering apoptosis and cell cycle arrest (21). However, in inflammation-driven cancers, IRF5 may promote tumor growth by chronic production of cytokines and immunological regulation (62).

### 4.3 IRF5 and antiviral immunity

RF5 is critical in initiating proinflammatory cytokine production via Toll-like receptor activation. Immune signaling in response to viral or bacterial infections typically induces type I IFN and inflammatory cytokine production, which helps eliminate pathogens. Upon viral invasion, IRF5 employs a dual mechanism: it promotes the synthesis of interferons (IFNs), powerful signaling molecules with antiviral properties, which in turn activate diverse immune cells to identify and destroy infected cells effectively (21, 63, 64). Studies reveal that Irf5^-/-^ mice show increased susceptibility to viral infections due to reduced IFN production and enhanced viral replication, highlighting IRF5’s essential role in antiviral defense (21). Additionally, emerging research suggests that IRF5’s antiviral functions extend beyond IFN production, potentially enhancing immune cell metabolism, particularly in macrophages, to strengthen antiviral responses (65, 66).

In the context of HIV infection, IRF5 plays a pivotal role by inducing various interferons that inhibit viral processes such as entry, replication, and assembly. IRF5 also stimulates the expression of antiviral genes, including PKR and 2’,5’-oligoadenylate synthetase, which aid in suppressing HIV replication (67), Specific IRF5-TNPO3 polymorphisms, such as rs2004640, rs10954213, rs2280714, and rs10279821, have been linked to enhanced HIV control in long-term non-progressors (LTNPs), suggesting these variants may boost IRF5’s antiviral activity and help maintain lower viral loads (68). However, recent findings indicate that IRF5 may predispose memory CD4+ T cells to Fas-mediated apoptosis and is integral to the Fas/FasL pathway; blocking this pathway with IRF5 inhibitory peptides has shown promise in preventing memory CD4+ T cell loss in HIV patients (69).

Elevated IRF5 expression and tissue damage are hallmarks of chronic inflammatory responses, suggesting that IRF5’s role in HIV-1 infection may extend to other chronic viral infections, including SARS-CoV-2 (64). COVID-19’s hyperinflammatory syndrome—often manifesting one to two weeks post-symptom onset—is termed “macrophage activation syndrome” (70), “cytokine storm” (71) or “acute respiratory distress syndrome” (72). This condition is characterized by excessive proinflammatory cytokines, chemokines (73), and other bioactive molecules, contributing to COVID-19 severity and mortality.

The proposed mechanism suggests that SARS-CoV-2, upon cell entry, may amplify the Hexosamine Biosynthetic Pathway (HBP), leading to increased glucose consumption and rapid viral replication. This heightened HBP activity raises levels of O-GlcNAc transferase (OGT), an enzyme with high binding affinity to IRF5 (74), potentially driving IRF5 overexpression. This overexpression can upregulate inflammatory cytokine genes and provoke detrimental endoplasmic reticulum (ER) stress, resulting in hyperinflammation, cytokine storm, and multiorgan failure (75).

Additionally, SARS-CoV-2 infection reduces IRF5+ myeloid dendritic cells (mDCs), although IRF5+ plasmacytoid dendritic cells (pDCs) remain unaffected (76). The IRAK4-IRF5 pathway significantly contributes to the hyperinflammatory cytokine and chemokine response in COVID-19 (77, 78). IRAK4 inhibitors reduce SARS-CoV-2-induced cytotoxicity in ACE2+ HEK293 cells by targeting IRF5 and IRF7, alongside reducing monokines and cytokines such as IL-6, IL-8, TNF-α, and CCL2, underscoring IRF5’s role in COVID-19 pathogenesis (78, 79).

### 4.4 IRF5 role in inflammatory bowel diseases

IRF5 has been widely studied in mouse models of colitis and IBD, and it is reported that it plays an important role in the inflammation of the colon. Research related to IRF5 knockout mice (IRF5^-^/^-^) has revealed significant protection against experimental colitis, which includes DSS- and TNBS-induced colitis, mainly attributed to decreased pro-inflammatory polarization of macrophages and reduced levels of cytokines including IL-6, IL-12 and TNF-α (6, 80). These results indicate that IRF5 is an important regulator of the response to inflammation in colitis and a prospective therapeutic target for inflammatory bowel disease (IBD). Nonetheless, transforming these results into therapeutic implications needs further evidence via human studies.

Various research investigated patient specimens to study IRF5’s role in human IBD. IRF5 polymorphisms have been related to an increased vulnerability to IBD, specifically Crohn’s disease (CD) and ulcerative colitis. For example, particular IRF5 risk alleles have been linked to greater disease severity and inflammatory cytokine production in IBD patients’ colon biopsies (81). Furthermore, IRF5 mRNA and protein levels have been reported to be higher among monocytes and colonic macrophages in IBD patients than in healthy controls, indicating that it plays a role in disease pathophysiology (80). These outcomes are consistent with mouse studies and support the involvement of IRF5 in its role as pro-inflammatory mediator in IBD and colitis.

While preclinical evidence clearly justifies focusing on IRF5 for IBD therapy, comprehensive clinical trials testing IRF5 antagonists in patients with IBD are currently missing. Emerging methods for autoimmune diseases, such as small-molecule antagonists and antisense oligonucleotides inhibiting IRF5 signaling, are currently under development and could lead the pathway for further clinical trials in IBD (46). Furthermore, given IRF5’s contribution to macrophage polarization, IRF5-targeted therapies may provide a more specific approach to modifying colon immune response while maintaining protective immunity. Overall, whereas human samples provide strong evidence supporting IRF5 as a viable option for therapy in IBD, more clinical research and treatment interventions are needed to demonstrate its translational significance.

### 4.5 IRF5 and tumor progression

IRF5 has been identified as a mediator of DNA damage-induced apoptosis and as a regulator of cell migration (21, 23, 82), suggesting that its loss may enhance cell proliferation and migration—traits consistent with cancer hallmarks (83), such as sustained proliferative signaling, invasion, metastasis, and resistance to cell death. Thus, IRF5 dysregulation may contribute to tumor initiation, progression, and metastasis, as well as impact treatment response (84). Extensive evidence shows IRF5 can function as either a tumor suppressor or a proto-oncogene, depending on cell type and tissue specificity.

Research indicates that IRF5 may act as a tumor suppressor, where its loss promotes tumor growth, metastasis, and poor outcomes in cancers such as breast, gastric, colorectal, pancreatic, and lung (82, 85–88). For example, IRF5 expression declines significantly in advanced ductal carcinoma *in situ* (DCIS) and is nearly absent in invasive ductal carcinoma, with low levels correlating to poorer prognosis in ER/PR-negative breast and non-small cell lung cancers (89–91). High IRF5 expression is linked to improved survival in pancreatic, head and neck squamous, and laryngeal cancers (85, 92, 93). The mechanisms responsible for reduced IRF5 expression in cancer remain largely unclear. A preliminary analysis of TCGA genomics data indicates that IRF5 rarely undergoes gene mutations or loss of heterozygosity. Thus, the reduced expression of IRF5 in tumors is likely attributable to expression loss or inhibition through post-transcriptional modifications rather than gene mutations (84).

The loss of IRF5 drives tumorigenesis through multiple mechanisms, primarily manifested as dysregulation of cell cycle control and apoptosis, activation of oncogenes, immune evasion, and enhanced cell migration and invasion abilities. In cell cycle regulation, IRF5 deficiency impedes the expression of genes such as Bak, caspase 8, Bax, and p21, preventing cells from entering a stagnation state or undergoing apoptosis, thus acquiring the ability for sustained proliferation (21, 23). At the same time, the loss of IRF5 is closely associated with the overexpression of the MYC gene, which not only promotes the generation of cancer stem cells but also correlates with reduced immune cell infiltration and decreased chemotherapy responses. In immune regulation, IRF5, as a core regulator of type I IFN and pro-inflammatory cytokine expression, loses its function, weakening the anti-tumor immune response in the tumor microenvironment, promoting dysregulated angiogenesis and inflammation, and creating conditions for tumor immune evasion (94). Moreover, IRF5 deficiency also leads to dysregulation of epithelial-mesenchymal transition (EMT), significantly enhancing the migration and invasion abilities of tumor cells, and making them more likely to form metastases (95). Notably, the function of IRF5 involves not only transcriptional regulation but also the limitation of cell migration and invasion through the regulation of cytoskeletal molecules (82). Its loss disrupts this crucial mechanism, further promoting tumor progression.

Moreover, IRF5 can also act as a proto-oncogene, with elevated levels in tumors like hepatocellular carcinoma (HCC) (96), non-metastatic clear cell renal carcinoma (97), endometrial, prostate cancers (98), and primary central nervous system (CNS) tumors (99). While the exact mechanism remains unknown. Study shows IRF5 is overexpressed in HCC and promotes the proliferation and tumorigenic potential of HCC cells by upregulating the expression of lactate dehydrogenase A (LDHA) and promoting glycolysis (96). High IRF5 levels correlate with increased recurrence and poorer outcomes in prostate cancer (100) and accelerated proliferation in thyroid cancer cells (101). Overall, IRF5’s role as a tumor suppressor or proto-oncogene is context-dependent, varying by cell type and tissue (84).

However, in malignancies characterized by inflammation, such as colorectal and pancreatic cancers, IRF5 may contribute to tumor growth by maintaining a chronic inflammatory state. Research demonstrates that IRF5 controls the secretion of pro-inflammatory cytokines such as TNF-α, IL-6, and IL-12, which might result in tumor-associated inflammation and immune evasion (41, 101). In these situations, chronic activation of IRF5-driven inflammation may result in enhanced angiogenesis, tumor cell survival, and resistance to immunotherapy. Furthermore, IRF5 activation in tumor-associated macrophages has been linked to an inflammatory tumor microenvironment that promotes cancer cell proliferation and metastasis (82). These data indicate that, while IRF5 activation may be advantageous in some tumors, it could be a useful therapeutic strategy in others where persistent inflammation drives malignancy.

Depending on the tumor type and immunological environment, IRF5’s dual nature emphasizes the importance of a proficient strategy when targeting this transcription factor for cancer therapy.

### 4.6 IRF5 and ischemia diseases

Cerebral ischemia initiates a complex inflammatory response, which, while aiding in cell repair, can also exacerbate disease progression. Microglia, as the brain’s resident immune cells, are pivotal in triggering and sustaining post-stroke inflammation, positioning them as central players in this process. Therefore, regulating microglial activation post-stroke holds potential for improving stroke outcomes. Recent findings indicate that central IRF4-IRF5 signaling drives microglial activation and stroke prognosis more significantly than the peripheral IRF4-IRF5 axis in cerebral ischemia (102–104). Conditional IRF5 knockout (CKO) promotes M2 activation, reduces proinflammatory responses, and improves outcomes in cerebral ischemia, spinal cord ischemia/reperfusion (I/R), and neonatal hypoxic ischemic encephalopathy (HIE) models (105–107). Conversely, IRF5 upregulation enhances M1 activation, intensifies proinflammatory responses, and worsens outcomes (102, 105).

Additionally, TLR7/MyD88/IRF5 signaling aggravates myocardial ischemia-reperfusion (I/R) injury (108). Silencing IRF5 with siRNA *in vivo* accelerates inflammation resolution, aids wound and infarct healing, and reduces post-myocardial infarction heart failure, as shown by fluorescence molecular tomography and cardiac MRI (109). In liver ischemia, elevated TLR4/IRF5 mRNA and downstream cytokines were observed three hours post-reperfusion. Pretreatment with N-acetylcysteine, an antioxidant and anti-inflammatory agent, notably reduced TLR4/IRF5 mRNA and cytokine levels, thereby alleviating hepatic injury (110).

### 4.7 IRF5 and other diseases

Studies have also identified IRF5 as a key factor in diseases such as asthma (111, 112), metabolic diseases (113), vascular diseases (114–116), neuropathic pain (117, 118), and liver fibrosis (119, 120). For instance, severe asthma (SA) is a life-threatening condition often resistant to corticosteroids. Bronchoalveolar lavage (BAL) cells from severe asthmatic patients show elevated IRF5 expression compared to those from individuals with milder asthma or healthy controls. In an SA model, IRF5 knockout mice exhibited reduced IFN-γ and IL-17 responses and an enhanced response to corticosteroids, which suppressed the elevated Th2 response (121).

In summary, these findings highlight IRF5 as a promising therapeutic target, with growing interest in its potential for innovative treatments.

## 5 Strategies for regulating IRF5

As is known, IRF5 plays an important role throughout the immune system network and factors affecting the IRF5-meidated signaling pathway are numerous and complex, making it challenging to define an effective pathway to target IRF5. Specific strategies to target IRF5 included regulation of gene expression, inhibiting the activation of IRF5, and inhibiting the production of active dimers.

### 5.1 Regulation of IRF5 expression

**Figure 1:**
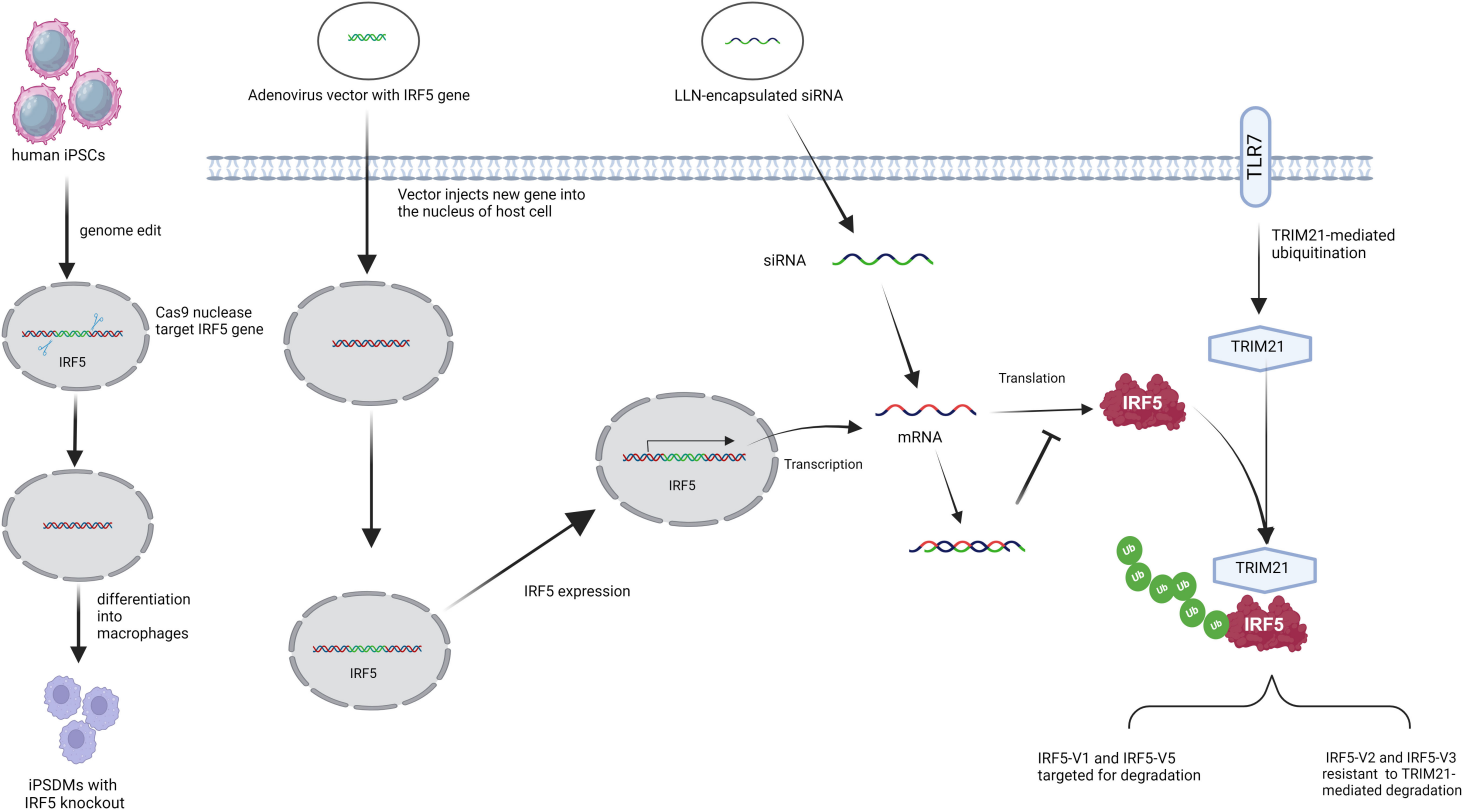
Strategies to regulate the expression of IRF5. (From left to right) IRF5 knockout in human-induced pluripotent stem cell-derived macrophages using the CRISPR/Cas9 system. Adenoviral vectors delivered IRF5 genes to increase IRF5 expression. IRF5 expression was modulated by RNA interference (RNAi) using small interfering RNA (siRNA) -mediated therapy delivered by lipid-like nanoparticles (LLN). After stimulation with Toll-like receptor 7 (TLR7), TRIpartite motif 21 (TRIM21) regulates IRF5 stability and activity in an isotype-specific manner.

Strategies to regulate IRF5 expression include small interfering RNA (siRNA), CRISPR-Cas9, and locked nucleic acid (LNA) oligonucleotides (ODNs) (**Figure 1**). Among these, siRNA targeting IRF5 mRNA is widely utilized, with studies demonstrating that lipid-like nanoparticles (LLNs) delivering IRF5-specific siRNA can mitigate post-myocardial infarction inflammation and promote tissue repair (109).

CRISPR-Cas9 has shown additional potential, as recent work using this technology to knock out IRF5 in induced pluripotent stem cell (iPSC)-derived macrophages resulted in reduced resistance to Chlamydia infection, underscoring IRF5’s role in anti-chlamydial immunity (**Figure 1**) (122).

The E3 ubiquitin ligase TRIM21 (**Figure 1**) also modulates IRF5 levels, targeting degradation primarily of IRF5 isotypes V1 and V5 after TLR7 activation, while sparing isotypes V2 and V3. V1 is mainly found in primary human plasmacytoid dendritic cells, and V5 is prevalent in peripheral blood mononuclear cells (9). Enhancing TRIM21 activity could help regulate immune responses by decreasing IRF5 levels in certain patients, though it may be less effective in V2-associated SLE cases (8, 123).

In contrast, targeted IRF5 overexpression has therapeutic potential. An adenoviral vector delivering IRF5 to the lungs enhanced immune responses following allergen exposure, reducing goblet cell proliferation, mucus production, and improving airway hyperresponsiveness. Additionally, IRF5 overexpression lowered IL-2 levels and eosinophil counts, making localized adenoviral delivery a promising approach for managing eosinophilic asthma without the side effects of systemic IRF5 expression (124, 125).

Collectively, these studies provide insights into therapeutic targeting of IRF5; however, significant challenges remain before these approaches are ready for clinical application.

### 5.2 Modulation of IRF5 activation

Phosphorylation activates IRF family members by inducing a conformational shift in their C-terminal autoinhibitory region, prompting dimerization (15). This underscores the importance of targeting kinases responsible for serine phosphorylation in IRF5 activation.

Multiple kinases, including RIP2, TAK1, and members of the IKK family (IKKα and IKKβ), can phosphorylate IRF5 (126–129). However, due to these kinases’ broader roles in other signaling pathways, targeting them for specific IRF5 inhibition is challenging. A novel small-molecule compound, YE6144, has been shown to selectively inhibit IRF5 phosphorylation and nuclear translocation, while only minimally affecting NF-κB activity in human PBMCs. Treatment with YE6144 reduced autoantibody production and slowed disease progression in NZB/W F1 mice, a model for systemic lupus erythematosus (SLE) (46).

IRAK4 has been identified as a kinase that activates IRF5 through the IRAK4-TAK1-IKKβ pathway in human monocytes. Selective inhibition of IRAK4 (using IRAK4 inhibitors or IRAK4i) disrupts IRF5 nuclear translocation and transcriptional activity without impacting NF-κB (18, 130). Recent studies indicate that IRAK4 inhibition can reduce inflammation and joint damage in rheumatoid arthritis (RA) by rebalancing macrophage and fibroblast metabolism, effectively decreasing RA disease activity (60, 131) Additionally, IRAK4 inhibition mitigates SARS-CoV-2-induced cytotoxicity in ACE2+ HEK293 cells (77, 78). and protects neurons by suppressing microglial inflammation following ischemic injury (132). These findings support IRAK4 as a promising therapeutic target.

### 5.3 IRF5 agonists/antagonists in clinical trials

Preclinical studies have found some possible IRF5 inhibitors that show potential in animal models, notably for autoimmune illnesses like systemic lupus erythematosus (SLE). The following is a summary of significant preclinical experiments (**Table 1**).

**Table 1:** Therapeutic strategies targeting IRF5 in preclinical models.

Research	Strategy	Model	Conclusion
Inhibition of IRF5 Hyperactivation Protects from Lupus Onset and Severity (51)	The production of peptide mimetics that specifically attach to inactive IRF5 monomers, blocking activation.	MRL/lpr, pristane-induced lupus animal models, and NZB/W F1	Treatment improved lupus pathology by lowering serum dsDNA titers, antinuclear autoantibodies, and circulating plasma cells, which improved renal pathology and prognosis.
*In Vivo* Silencing of the Transcription Factor IRF5 (109)	Utilizing siRNA (small interfering RNA) to inhibit cardiac macrophages’ production of IRF5	Myocardial infarction (MI) mouse model	Blocking IRF5 improved infarct healing and attenuated post–myocardial infarction remodeling, indicating potential therapeutic applications beyond autoimmune diseases.
Genetic and Chemical Inhibition of IRF5 Suppresses Pre-existing Systemic Autoimmunity (46)	Pharmacological and genetic blocking of IRF5 decreases pre-existing systemic autoimmunity.	A mouse model with Y-linked autoimmune accelerator (Yaa) and FcγRIIB deficiency.	Partially blocking IRF5 was more effective than full suppression of type I interferon signaling in reducing ailments, implying possible approaches to treatment for SLE.

These preclinical studies are promising, indicating that targeting IRF5 may constitute a potential treatment strategy for a variety of autoimmune and inflammatory diseases. However, further research is required to create IRF5-targeted treatments acceptable for human clinical trials.

### 5.4 Interference with IRF5‐interacting partners

#### 5.4.1 Peptide inhibitors targeting IRF5

**Figure 2:**
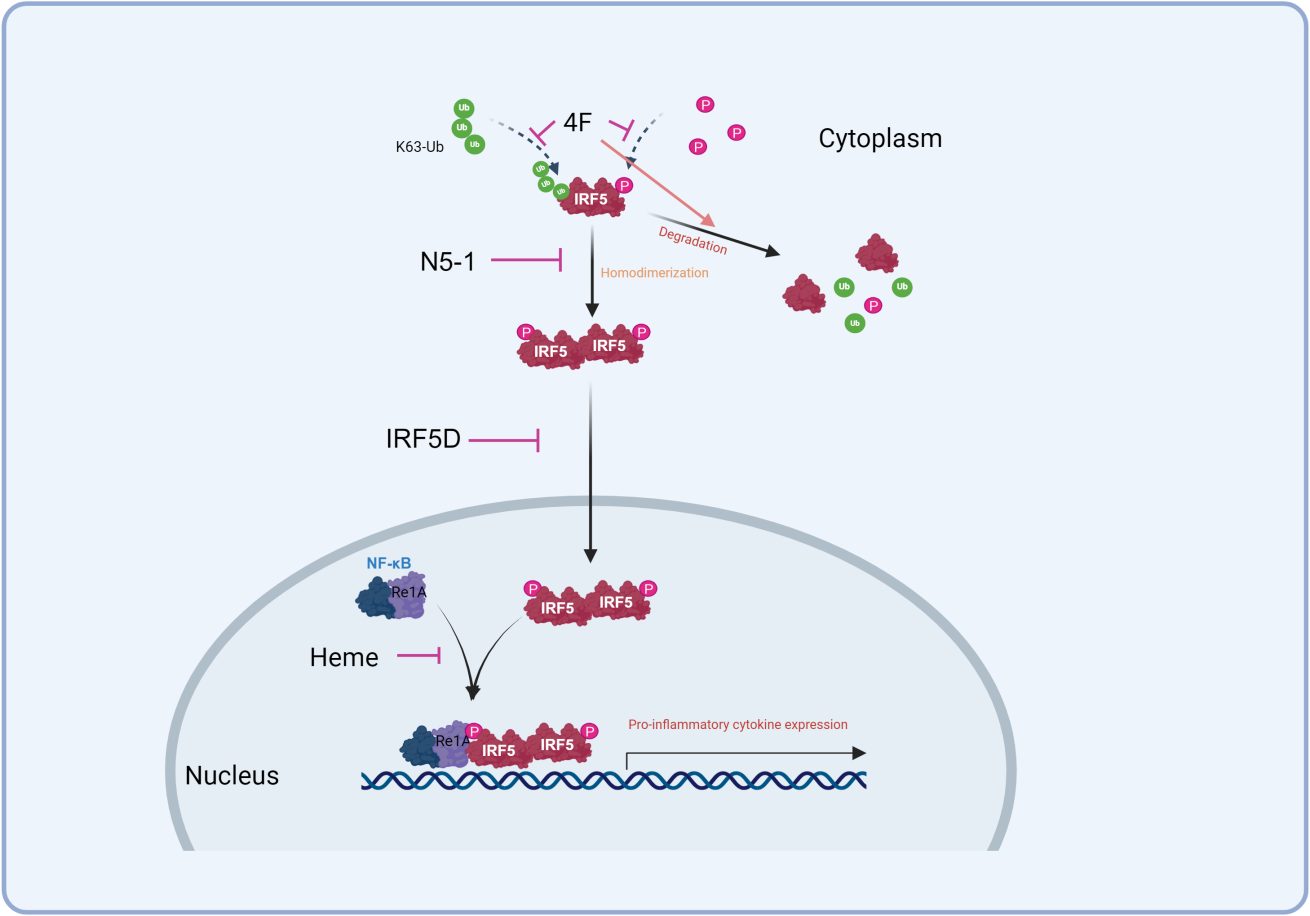
Strategies to inhibit IRF5. 4F competed for access to IRF5 to inhibit its activation and promote its degradation. N5-1 stabilizes the inactive monomer, inhibiting its homodimerization and nuclear translocation. IRF5D targets IRF5 and prevents its nuclear translocation. Heme inhibits the IRF5/RelA interaction to down-regulate inflammation.

Given IRF5’s extensive protein and DNA interaction regions, peptide inhibitors could serve as effective blockers of protein-protein interactions. However, their clinical application is challenged by low conformational stability, which diminishes binding efficacy (133). In a mouse model of systemic scleroderma, the apoA-I mimetic peptide 4F reduced pro-inflammatory HDL levels and competitively inhibited IRF5 activation, effectively reducing myocardial inflammation (**Figure 2**). This study also linked immune cell composition to varying inflammation levels (37).

Another promising inhibitor, IRF5D, was engineered to prevent IRF5’s nuclear translocation. Derived from IRF5’s C-terminal dimerization domain, IRF5D functions as a decoy by replacing the original target sequence with a 17-amino acid peptide. In a Tsk+/+ mouse model of myocardial inflammation and fibrosis, IRF5D treatment decreased ICAM-1 and IRF5 expression, reduced leukocyte infiltration, and improved vascular endothelial relaxation. These findings, supported by *in vitro* results, highlight IRF5 as a potential target for managing myocardial inflammation and fibrosis (115, 134). The IRF5D structure sets the foundation for developing future IRF5-specific peptide inhibitors (**Figure 2**) (15). Furthermore, a new peptide inhibitor, N5-1, shows strong affinity for IRF5, binding to and stabilizing the inactive monomer to prevent nuclear translocation. In lupus-prone mice, N5-1 demonstrated protective effects (**Figure 2**) (51, 135).

#### 5.4.2 Targeting IRF5 protein-protein interactions

IRF5 is recruited to the TNF-α gene through interaction with the NF-κB subunit RelA, which is essential for activating pro-inflammatory gene expression involving IRF5 (11, 32, 136). Blocking this IRF5-RelA interaction has emerged as a key therapeutic target (11, 137). Specific peptide inhibitors have been developed to spatially inhibit the IRF5/RelA interaction, thereby modulating inflammation. Studies have shown that heme can protect the intestinal mucosal barrier in DSS-induced colitis by regulating macrophage polarization via disruption of the IRF5-RelA complex (**Figure 2**) (138, 139). Other inhibitors, such as Sedum sarmentosum Bunge extract, Euphorbia Factor L2, and Lipoxin A4, also downregulate IRF5 and RelA activity to reduce inflammation (140–142).

Additionally, the chaperone CSN, specifically its subunit CSN3, binds directly to IRF5, enhancing its stability and transcriptional activity. CSN3 knockdown leads to IRF5 degradation, highlighting the CSN-IRF5 interaction as crucial for IRF5 activation and suggesting it as a potential target for disrupting IRF5 function (143). Targeting these protein binding sites on IRF5 could guide the development of peptide-based inhibitors, emphasizing the need to identify precise IRF5 binding sequences.

The Src kinase family member Lyn also negatively regulates IRF5 through the TLR-MyD88 pathway in a kinase-independent manner. Expressed in immune cells such as monocytes, macrophages, and B cells, Lyn interacts with IRF5, inhibiting its post-translational modifications. Lyn deficiency leads to IRF5 overactivation, exacerbating SLE-like symptoms in mice, while IRF5 deficiency alleviates these symptoms, supporting Lyn’s regulatory role in SLE (46, 144). Interestingly, IRF5’s role differs in asthma (145), where it enhances responses to allergens, modulating airway hyperresponsiveness, mucus secretion, and eosinophilic inflammation. Lyn peptide inhibitors block eosinophil differentiation and airway infiltration in asthma models, indicating that Lyn-IRF5 interaction inhibitors might enhance IRF5’s protective role in asthma (125, 146–148).

Oligodeoxynucleotides (ODNs) mimicking DNA’s immunosuppressive properties are also under investigation as IRF5 inhibitors. The ODN MS19, containing an AAAG-rich sequence that binds IRF5, reduces nuclear translocation and expression of inflammatory markers (iNOS, IL-6, and TNF-α). MS19 has shown efficacy in models of septic peritonitis, acute lung injury (ALI), and myocarditis, alleviating systemic inflammatory responses (149–152). Furthermore, MS19 could attenuate systemic inflammatory responses and decrease IRF5 expression in burn injury skin and myocardial tissues of coxsackie virus B3-infected mice (150, 153). Additionally, MS19’s inhibitory effect on LPS-induced ALI appears to act through the HMGB1-TLR4-NF-κB pathway (154), supporting its potential as a therapeutic target for inflammatory conditions.

While these findings underscore IRF5 as a promising therapeutic target, significant challenges remain in translating these methodologies into clinical applications.

## 6 Summary

This review highlights the critical roles of IRF5 in various diseases and explores its potential as a therapeutic target. Strategies to regulate IRF5 activity and expression are examined, including approaches to modulate IRF5 levels, disrupt post-translational modifications, and inhibit its interactions with protein chaperones. Emerging therapies such as siRNAs, nanoparticles, CRISPR/Cas9, and adenoviral vectors show promise but require further development to ensure clinical feasibility. At present, kinase inhibitors represent a particularly promising strategy for specifically inhibiting the IRF5 pathway. Ideal kinase inhibitors should target IRF5 selectively, avoiding off-target effects; inhibitors affecting broader pathways (e.g., TAK1 or IKKβ) risk unwanted side effects due to lack of specificity. IRAK4 inhibitors, however, exhibit specific selectivity for the IRF5 pathway and have shown therapeutic potential in RA, ischemia, and SARS-CoV-2 infection models.

Additionally, YE6144 selectively inhibits IRF5 phosphorylation, demonstrating strong therapeutic promise. Lyn peptide inhibitors have shown benefit in asthma models, though further safety evaluations are warranted given potential risks like lupus associated with Lyn suppression. Notably, the novel inhibitor N5-1 binds IRF5 and blocks its nuclear translocation, displaying protective effects in lupus-prone mice. Similarly, small-molecule inhibitors targeting IRF5 interactions with RelA or CSN could suppress pro-inflammatory macrophage activation by inhibiting IRF5’s downstream targets, though further research is needed.

In summary, modulating IRF5 presents a compelling avenue for developing new treatments for inflammatory diseases, tumors, and certain autoimmune disorders.
